# Association of the miR-17-5p variants with susceptibility to cervical cancer in a Chinese population

**DOI:** 10.18632/oncotarget.12299

**Published:** 2016-09-28

**Authors:** Tianbo Jin, Xiaohong Wu, Hua Yang, Ming Liu, Yongjun He, Xue He, Xugang Shi, Fengjiao Wang, Shuli Du, Yajuan Ma, Shan Bao, Dongya Yuan

**Affiliations:** ^1^ Key Laboratory of Molecular Mechanism and Intervention Research for Plateau Diseases of Tibet Autonomous Region, School of Medicine, Xizang Minzu University, Xianyang, Shaanxi 712082, China; ^2^ Key Laboratory of High Altitude Environment and Genes Related to Diseases of Tibet Autonomous Region, School of Medicine, Xizang Minzu University, Xianyang, Shaanxi 712082, China; ^3^ Key Laboratory for Basic Life Science Research of Tibet Autonomous Region, School of Medicine, Xizang Minzu University, Xianyang, Shaanxi 712082, China; ^4^ Xi'an Tiangen Precision Medical Institute, Xi'an, Shaanxi 710075, China; ^5^ Department of Maternity care, Xi'an Maternal and Child Health Hospital, Xi'an, Shaanxi 710002, China; ^6^ Department of Obstetrics and Gynecology, Second Affiliated Hospital, Xi'an Jiaotong University, Xi'an 710004, China; ^7^ Clinic of Gynecology and Obstetrics, Hainan Provincial People's Hospital, Haikou, 570102, China

**Keywords:** cervical cancer, case-control studies, miR-17-5p, single nucleotide polymorphisms (SNPs)

## Abstract

MicroRNAs (miRNAs) are key regulators of gene expression; however, the extent to which single nucleotide polymorphisms (SNPs) interfere with miRNA gene regulation and affect cervical cancer (CC) susceptibility remains largely unknown. Here, we systematically analyzed miRNA-related SNPs and their association with CC risk, and performed a case-control study of miR-17-5p SNPs and CC risk in a Chinese population. Sixteen SNPs were genotyped in 247 CC cases and 285 controls. Three were associated with CC risk (*p* < 0.05): the minor allele (A) of rs217727 in H19 increased risk (OR = 1.53, *p* = 0.002), while the minor alleles (T) of rs9931702 and (T) of rs9302648 in *AKTIP* decreased CC risk (*p* = 0.018, *p* = 0.014). Analysis of the SNPs after stratification based on CC clinical stage and subtype revealed that rs1048512, rs6659346, rs217727, rs9931702, and rs9302648 were associated with CC risk in clinical stages I-II; rs2862833, rs2732044, rs1030389, and rs1045935 were associated with CC risk in clinical stages III-IV; and rs217727, rs9931702, and rs9302648 were associated with CC risk in squamous carcinomas. These data could serve as a useful resource for understanding the miR-17 function, identification of miRNAs associated with CC, and development of better CC screening strategies.

## INTRODUCTION

Cervical cancer (CC) is the second most common cancer among women worldwide [1, 2]. Even though more than 95% of CC patients are infected with high-risk types of human papillomavirus (HPV), HPV infection alone is not sufficient to cause cervical cancer [3–5]. Cervical cancer is thought to be a multifactorial and complex disease that results from the interaction between heredity and environment. In addition, epidemiological studies have suggested that host genetic variations may contribute to CC pathogenesis [6].

MicroRNAs (miRNAs) are small, endogenous, non-coding RNA molecules, which are about 21–23 nucleotides in length, and regulate gene expression by translational repression or mRNA degradation [7]. MiRNAs are involved in various biological processes, including cell proliferation, cell death, and fat metabolism [8]. The miR-17-5p originates from the mature microRNA gene product of the miR-17 gene precursor 5p. Abnormal expression of miR-17-5p has been observed in different types of cancer, including lung [9, 10], pancreatic [11], liver [12], breast [13], gastric [14], and cervical cancer [15], suggesting that miR-17- 5p may have an important role in cancer occurrence and development.

Single nucleotide polymorphisms (SNPs) are important variations determining the diversity among individuals; they cause different phenotypes, traits, and diseases [16]. Since miRNAs are key regulators of gene expression, miRNA-related SNPs including SNPs in miRNA genes and target sites may affect phenotypes and disease susceptibility [17]. Moreover, SNPs located in miRNAs are likely to affect miRNAs maturation, functional strand selection, and target selection. To date, a number of studies have demonstrated that SNPs in target sites or miRNA genes are associated with various diseases [17–19]. For example, the functional SNP in pre-miR-146a may contribute to esophageal squamous cell carcinoma susceptibility and clinical outcome. miR-196a-2 might have an oncogenic role in breast tumorigenesis, and the functional genetic variant in its mature region could serve as a novel biomarker for breast cancer susceptibility.

In this study, we performed a screening for common SNPs in miR-17-5p sequence and evaluated 16 SNPs with respect to their association with the susceptibility to cervical cancer in a Chinese population.

## RESULTS

Selected characteristics of 247 CC cases and 285 cancer-free controls are summarized in Table 1. Of the 247 CC cases, 149 had stage I-II carcinoma, and 91 cases had stage III-IV carcinoma. There were 88 squamous carcinoma cases, and 239 adenocarcinoma cases. The observed genotype distributions for the 16 SNP loci polymorphisms in controls and patients were consistent with the Hardy-Weinberg equilibrium (*p* > 0.05). Using the χ^2^ test, 3 SNPs were associated with an increased or reduced risk of cervical cancer (*p* < 0.05; Table 2). Among them, the minor allele (A) of rs217727 in the *H19* gene increased the risk (OR = 1.53, 95% CI, 1.17–2.02, *p* = 0.002), while the minor allele (T) of rs9931702, and the minor allele (T) of rs9302648 in the *AKTIP* gene decreased the risk of cervical cancer (OR = 0.73, 95% CI, 0.56–0.95, *p* = 0.018; OR = 0.72, 95% CI, 0.55–0.94, *p* = 0.014).

**Table 1:** Distributions of selected variables in CC cases

Characteristics		Cases
Number		247
Age	≤ 54	132
	> 54	115
Clinical stages	I- II	149
	III- IV	91
Subtype	squamous carcinomas	8
	adenocarcinoma	239

**Table 2:** Frequency distributions of alleles and their associations with cervical cancer

SNP	Chromosome	Band	Gene (s)	Alleles A/B	MAF (case)	MAF (control)	HWE-P	OR (95% CI)	*P*
rs1048512	1	1q23.2	PIGM	A/G	0.099	0.112	0.23	0.87 (0.59–1.29)	0.489
rs6659346	1	1q23.2	PIGM	A/G	0.111	0.118	0.15	0.94 (0.64–1.37)	0.751
rs11006369	10	10q11.23	SGMS1	T/A	0.121	0.093	0.72	1.35 (0.91–1.99)	0.133
rs2862833	10	10q23.31	FAS	G/A	0.490	0.456	0.15	1.14 (0.90–1.46)	0.272
rs3741216	11	11p15.5	H19	A/T	0.077	0.074	0.38	1.05 (0.66–1.65)	0.842
rs217727	11	11p15.5	H19	A/G	0.315	0.231	0.74	1.53 (1.17–2.02)	0.002
rs2839702	11	11p15.5	H19	C/A	0.356	0.384	0.13	0.88 (0.69–1.44)	0.337
rs2067051	11	11p15.5	MIR675	T/C	0.336	0.376	0.10	0.84 (0.65–1.08)	0.180
rs2274062	13	13q22.2	LMO7	A/T	0.453	0.498	0.15	0.84 (0.66–1.06)	0.145
rs9318375	13	13q22.2	LMO7	T/A	0.043	0.058	0.24	0.72 (0.41–1.27)	0.254
rs2732044	15	15q11.2	SNORD109B	A/G	0.427	0.442	0.90	0.94 (0.74–1.20)	0.623
rs1030389	15	15q11.2	SNORD109B	G/A	0.439	0.467	0.34	0.90 (0.70–1.14)	0.371
rs1045935	15	15q11.2	SNORD109B	G/T	0.540	0.486	0.72	1.24 (0.98–1.58)	0.076
rs12902710	15	15q21.3	PIGB	C/T	0.500	0.461	0.34	1.17 (0.92–1.49)	0.209
rs9931702	16	16q12.2	AKTIP	T/C	0.271	0.339	0.90	0.73 (0.56–0.95)	0.018
rs9302648	16	16q12.2	AKTIP	T/G	0.269	0.339	0.90	0.72 (0.55–0.94)	0.014

*p*- value ≤ 0.05 indicates statistical significance.

HWE, Hardy-Weinberg Equilibrium; MAF, minor allele frequency; ORs, odds ratios; CI, confidence interval.

As shown in Table 3, for rs2862833, the genotype “G/G” was associated with a decreased risk of cervical cancer in the dominant model (OR = 0.65, 95% CI, 0.46–0.92, *p* = 0.013). The logistic regression analysis revealed that the rs568408 GG genotypes compared with the AA/GA genotypes were associated with a significantly increased risk of cervical cancer in a recessive genetic model (OR = 1.66, 95% CI, 1.11–2.50, *p* = 0.014). Compared with the GG genotype, the AG and AA of rs217727 polymorphism in CC patients differed from the controls (AG vs GG, OR = 1.50, 95% CI, 1.05–2.16; AA vs GG, OR = 2.35, 95% CI, 1.21–4.57; *p* = 0.0097; AG/AA vs GG, OR = 1.62, 95% CI, 1.15–2.29, *p* = 0.0059). Similarly, variant rs9931702 TC genotypes were associated with a reduced CC risk (TC vs CC, OR = 0.66, 95% CI, 0.46 – 0.95, *p* = 0.045; TC/TT vs CC, OR = 0.65, 95% CI, 0.46 – 0.92, *p* = 0.013) compared with the wild-type rs3212227 CC. The genotype “C/T-T/T” of rs9302648 was associated with a decreased risk of cervical cancer in the dominant model (OR = 0.65, 95% CI, 0.46–0.92, *p* = 0.013), and in the codominant model (GT vs GG, OR = 0.67, 95% CI, 0.46 – 0.96, *p* = 0.042). No association was observed between the rest of the polymorphism and the CC risk. Using Bonfferroni correction, we have not found any SNPs to have a significant risk (*p* < 0.05/16*5).

**Table 3:** The associations between the gene polymorphisms of cervical cancer patients

	Model	Genotype	control	case	OR (95% CI)	*P*-value
rs2862833	Codominant	A/A	78 (27.4%)	73 (29.6%)	1	0.016
		G/A	154 (54%)	106 (42.9%)	0.74 (0.49–1.10)	
		G/G	53 (18.6%)	68 (27.5%)	1.37 (0.85–2.22)	
	Dominant	A/A	78 (27.4%)	73 (29.6%)	1	0.58
		G/A-G/G	207 (72.6%)	174 (70.5%)	0.90 (0.62–1.31)	
	Recessive	A/A-G/A	232 (81.4%)	179 (72.5%)	1	0.014
		G/G	53 (18.6%)	68 (27.5%)	1.66 (1.11–2.50)	
	Log-additive	---	---	---	1.14 (0.90–1.45)	0.28
rs217727	Codominant	G/G	169 (59.5%)	117 (47.6%)	1	0.0097
		A/G	99 (34.9%)	103 (41.9%)	1.50 (1.05–2.16)	
		A/A	16 (5.6%)	26 (10.6%)	2.35 (1.21–4.57)	
	Dominant	G/G	169 (59.5%)	117 (47.6%)	1	0.0059
		A/G-A/A	115 (40.5%)	129 (52.4%)	1.62 (1.15–2.29)	
	Recessive	G/G-A/G	268 (94.4%)	220 (89.4%)	1	0.036
		A/A	16 (5.6%)	26 (10.6%)	1.98 (1.04–3.78)	
	Log-additive	---	---	---	1.52 (1.16–1.99)	0.0023
rs9931702	Codominant	C/C	124 (43.5%)	134 (54.2%)	1	0.045
		T/C	129 (45.3%)	92 (37.2%)	0.66 (0.46–0.95)	
		T/T	32 (11.2%)	21 (8.5%)	0.61 (0.33–1.11)	
	Dominant	C/C	124 (43.5%)	134 (54.2%)	1	0.013
		T/C-T/T	161 (56.5%)	113 (45.8%)	0.65 (0.46–0.92)	
	Recessive	C/C-T/C	253 (88.8%)	226 (91.5%)	1	0.29
		T/T	32 (11.2%)	21 (8.5%)	0.73 (0.41–1.31)	
	Log-additive	---	---	---	0.73 (0.56–0.95)	0.019
rs9302648	Codominant	G/G	124 (43.5%)	134 (54.2%)	1	0.042
		G/T	129 (45.3%)	93 (37.6%)	0.67 (0.46–0.96)	
		T/T	32 (11.2%)	20 (8.1%)	0.58 (0.31–1.06)	
	Dominant	G/G	124 (43.5%)	134 (54.2%)	1	0.013
		G/T-T/T	161 (56.5%)	113 (45.8%)	0.65 (0.46–0.92)	
	Recessive	G/G-G/T	253 (88.8%)	227 (91.9%)	1	0.22
		T/T	32 (11.2%)	20 (8.1%)	0.70 (0.39–1.25)	
	Log-additive	---	---	---	0.72 (0.56–0.94)	0.015

*p*-value ≤ 0.05 indicates statistical significance.

**Figure 1:**
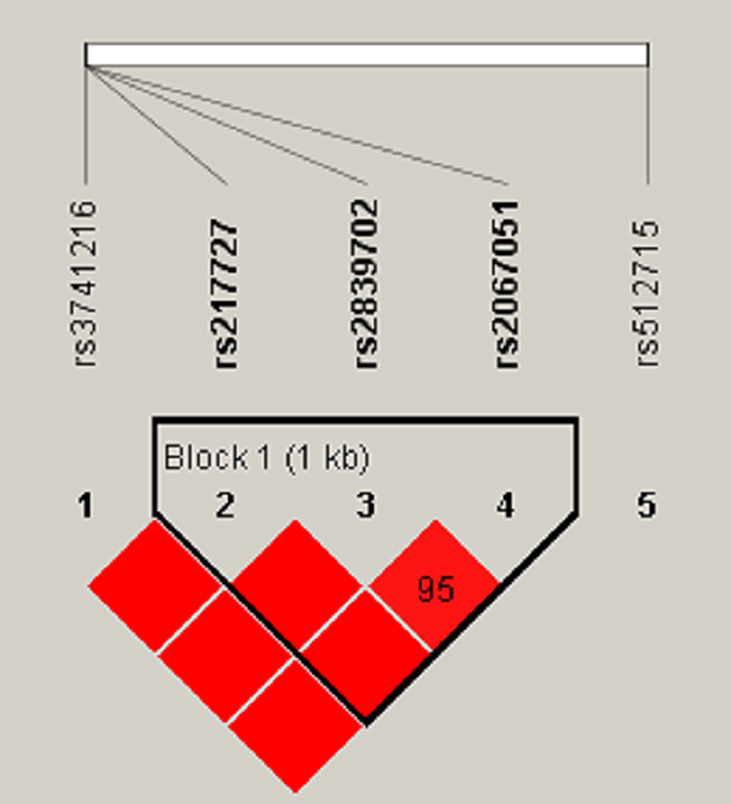
Haplotype block map for the SNPs genotyped in this study Brighter red represents stronger LD (LOD = 2, D′ = 1)

CI, confidence interval; ORs, odds ratios;

Linkage disequilibrium (LD) and haplotype analyses of the SNPs in CC patients and control samples were further studied (Figure 1). The results of the association between the H19 and AKTIP haplotype and the risk of cervical cancer are listed in Table 4. Compared with the GCT wild type, carriers of AAC (rs217727, rs2839702, rs2067051) haplotypes had significant associations with increased susceptibility to cervical cancer (OR = 1.51, 95% CI, 1.11 - 2.06, *p* = 0.0094). For the rs9931702 and rs9302648 in the AKTIP gene, compared with the TT wild type, carriers of CG haplotypes had significant associations with decreased CC risk (OR = 0.72, 95% CI, 0.56–0.94, *p* = 0.016).

**Table 4:** The haplotype frequencies of gene polymorphisms and cervical cancer risk

rs217727	rs2839702	rs2067051	Freq	OR (95% CI)	*P*-value
G	C	T	0.3509	1	---
G	A	C	0.3505	0.98 (0.75–1.29)	0.9
A	A	C	0.2704	1.51 (1.11–2.06)	0.0094
G	C	C	0.0203	1.28 (0.53–3.12)	0.58

rs217727	rs2839702	rs2067051	Freq (case)	Freq (control)	OR (95% CI)	*P*-value
G	C	T	0.334	0.366	1	---
G	A	C	0.324	0.374	0.98 (0.75–1.29)	0.9
A	A	C	0.316	0.231	1.51 (1.11–2.06)	0.0094
G	C	C	0.023	0.018	1.28 (0.53–3.12)	0.58

rs9931702	rs9302648	Freq	OR (95% CI)	P-value
C	G	0.6927	1	---
T	T	0.3064	0.72 (0.56–0.94)	0.016

rs9931702	rs9302648	Freq (case)	Freq (control)	OR (95% CI)	P-value
C	G	0.732	0.661	1	---
T	T	0.268	0.339	0.72 (0.56–0.94)	0.016

CI, confidence interval; ORs, odds ratios; *p*-value ≤ 0.05 indicates statistical significance.

Stratified analysis of the effect of these SNPs polymorphisms on cervical cancer by clinical stages and subtype is shown in Table 5. The results indicate that rs1048512, rs6659346, rs217727, rs9931702, and rs9302648 are associated with CC risk in clinical stages of I-II, while rs2862833, rs2732044, rs1030389, and rs1045935 are associated with CC risk in clinical stages of III-IV. Concerning subtypes, rs217727, rs9931702, and rs9302648 are associated with CC risk in squamous carcinomas, while there is no association of SNPs in adenocarcinoma.

**Table 5:** The associations between the gene polymorphisms and clinical characteristics of cervical cancer patients

SNP	clinical stages				subtype			
	I- II		III- IV		Squamous carcinomas		adenocarcinoma	
	OR (95%CI)	*p*			OR (95%CI)	*p*	OR (95%CI)	*p*
rs1048512	0.57 (0.34–0.96)	0.033	1.38 (0.85–2.24)	0.194	0.88 (0.59–1.31)	0.536	0.53 (0.07–4.06)	0.532
rs6659346	0.60 (0.36–0.99)	0.044	1.54 (0.97–2.45)	0.066	0.96 (0.65–1.4)	0.818	0.5 (0.07–3.85)	0.498
rs11006369	1.47 (0.95–2.28)	0.085	1.2 (0.7–2.07)	0.502	1.32 (0.89–1.96)	0.167	2.25 (0.62–8.15)	0.205
rs2862833	1.01 (0.77–1.34)	0.920	1.45 (1.04–2.03)	0.028	1.12 (0.87–1.42)	0.381	2.62 (0.9–7.65)	0.067
rs3741216	0.71 (0.39–1.29)	0.263	1.55 (0.89–2.72)	0.122	1.02 (0.64–1.63)	0.920	1.8 (0.39–8.17)	0.442
rs217727	1.81 (1.33–2.46)	0.000	1.23 (0.84–1.8)	0.289	1.55 (1.18–2.04)	0.002	1.11 (0.35–3.51)	0.856
rs2839702	0.86 (0.64–1.15)	0.308	0.86 (0.61–1.22)	0.409	0.88 (0.69–1.14)	0.331	0.96 (0.34–2.68)	0.940
rs2067051	0.76 (0.56–1.03)	0.073	0.92 (0.65–1.3)	0.623	0.84 (0.65–1.08)	0.170	1 (0.36–2.78)	0.994
rs2274062	0.77 (0.58–1.02)	0.067	0.99 (0.71–1.38)	0.930	0.83 (0.65–1.06)	0.134	1.01 (0.37–2.72)	0.989
rs9318375	0.98 (0.54–1.80)	0.959	0.37 (0.13–1.05)	0.051	0.71 (0.4–1.26)	0.237	1.08 (0.14–8.47)	0.938
rs2732044	1.10 (0.83–1.46)	0.494	0.7 (0.5–0.99)	0.043	0.93 (0.73–1.19)	0.571	1.26 (0.47–3.41)	0.646
rs1030389	1.04 (0.79–1.38)	0.783	0.68 (0.48–0.96)	0.028	0.89 (0.7–1.13)	0.341	1.14 (0.42–3.09)	0.792
rs1045935	1.07 (0.81–1.42)	0.627	0.6 (0.43–0.85)	0.004	0.79 (0.62–1)	0.053	0.63 (0.23–1.77)	0.381
rs12902710	1.25 (0.94–1.65)	0.121	1.02 (0.73–1.43)	0.895	1.15 (0.9–1.46)	0.267	1.95 (0.7–5.42)	0.196
rs9931702	0.68 (0.50–0.93)	0.015	0.87 (0.61–1.24)	0.441	0.75 (0.57–0.97)	0.029	0.28 (0.06–1.24)	0.074
rs9302648	0.68 (0.50–0.93)	0.015	0.85 (0.59–1.21)	0.363	0.74 (0.57–0.96)	0.024	0.28 (0.06–1.24)	0.074

CI, confidence interval; ORs, odds ratios; *p*-value ≤ 0.05 indicates statistical significance.

## DISCUSSION

The association between the SNPs and cancer risk has been investigated extensively. However, few studies concerning the SNPs in miRNA genes associated with cancer are available. In the present case-control study, we focused on the association between SNPs involved in the miR-17-5p with the cervical cancer risk in Chinese women. To the best of our knowledge, this is the first study to show miR-17-5p SNPs polymorphisms in cervical cancer. We have found that four SNPs (rs217727, rs2862833, rs9931702, and rs9302648) of 16SNPs in the miR-17-5p are associated with cervical cancer risk.

MicroRNAs are small endogenous noncoding RNAs that function as post-transcriptional regulators, and control genes involved in the regulation of cell cycle, proliferation, and cell death [20–22]. The miR-17-5p originates from the mature microRNA gene product of the miR-17 gene precursor 5p. The miR-17-92 gene cluster is a highly conserved gene cluster that encodes miR-17-5p, miR-17-3p, miR-18a, miR-19a, miR-20a, miR-19b-1 and miR-92-1 [23]. Expression of the miR-17-92 gene cluster is increased in several cancers, including colorectal, lung, pancreatic, and liver cancer. miR-17-5p acts as a tumor suppressor in prostate, cervical, and breast cancer. In addition, miR-17-5p and miR-20a are involved in c-Myc-modulated E2F1 expression. Inhibition of miR-17-5p and miR-20a in a cervical cancer cell line upregulates the E2F1 oncogene [24]. In addition, miR-17-5p and miR-20a alleviate the suppressive function of myeloid-derived suppressor cells by modulating STAT3 expression [25], and miR-17-5p functions as a tumor suppressor by targeting TP53INP1 in cervical cancer cells.

Our findings confirm the observations that rs217727 in *H19* gene is associated with cervical cancer. The *H19* gene is located in a cluster with the insulin-like growth factor 2 (IGF2) gene on chromosome 11p15.5. The *H19* gene does not encode for a protein, but instead codes for a capped, spliced, and polyadenylated 2.7-kb RNA that plays important roles in embryonic development and growth control [26]. However, *H19* expression is reduced after birth, and its expression is only found in cardiac and skeletal muscle. Increasing evidence suggests that *H19* is abnormally expressed in breast, liver, lung, cervical, esophageal, and bladder tumors [27, 28], and promotes cancer cell proliferation, suggesting an oncogenic function. The C/T polymorphism rs217727 is located in exon 5 of the *H19* gene. Although the rs217727 polymorphism (C > T) does not affect H19 mRNA expression levels, mutation may alter the translational efficiency, potentially leading to alterations in H19 structure, which may ultimately influence the function of *H19*. *H19* is abnormally expressed in several tumors, and acts as either a tumor suppressor [28, 29], or an oncogene [30]. Increasing evidence suggests that *H19* genetic variants play important roles in cancer development as well as other diseases. For example, Gao et al. [31] found that rs217727C to T variant was associated with increased coronary artery disease risk (CAD). In another study, maternal *H19* rs217727 TT genotype was associated with a higher birth weight. A previous study by Yang et al. [32] evaluated the effects of 4 independent SNPs (*H19* rs217727, rs2839698, rs3741216, and rs3741219) on gastric cancer risk in the Chinese population and suggested that the T allele of rs217727 was associated with higher risk of gastric cancer.

Rs9931702 and rs9302648 are located in the *AKTIP* gene. AKT-interacting protein (AKTIP) is a cell membrane protein that interacts with protein kinase B (PKB)/Akt and modulates PKB activity by enhancing PKB's phosphorylation and interaction with the upstream kinase phosphoinositide-dependent kinase 1 (PDK1). However, the exact biological function of *AKTIP* is not fully understood. To our knowledge, this is the first study evaluating the *AKTIP* function and its association with cervical cancer risk.

We stratified analysis of the effect of these SNPs polymorphisms on cervical cancer by subtype. The results indicated that there was no same SNPs have significance in different subtypes. The phenomenon suggested that the pathogenesis of CC different organizations vary. This played an important role for us to study the clinical pathological features and clinical guide and in predicting prognosis.

These data will Our results should serve as a useful resource for understanding the miR-17 function and identification of miRNAs associated with cervical cancer. Better understanding of the miR-17-5p function and identification of other CC-associated miRNAs will increase our understanding of the CC etiology and provide new strategies for CC screening.

## MATERIALS AND METHODS

### Study participants

This case-control study included 247 patients with cervical cancer and 285 healthy controls. All CC cases were newly diagnosed, histologically confirmed CC patients consecutively recruited between 2015 and 2016, from Second Affiliated Hospital, Xi'an Jiaotong University. Patients' evaluations were performed by trained medical personnel as per the guidelines outlined by the International Federation of Gynecology and Obstetrics (FIGO). Patients who had history of cancer, chromosomal abnormalities, or radiotherapy or chemotherapy were excluded from the study. The cancer-free control group consisted of women in good health and with no malignancy history, who were recruited during regular gynecological examinations. All women were interviewed and given an epidemiological questionnaire that included age, ethnicity, residential region, diet, and family history of cervical cancer. All subjects were genetically unrelated Chinese Uighur. After informed consent was obtained, 5 ml of peripheral blood sample was collected from each subject. This study was approved by Second Affiliated Hospital, Xi'an Jiaotong University and Xizang Minzu University.

### SNP selection and genotyping

SNPs were selected based on published studies demonstrating an association with cancer [27–28, 31–33], and by using search of HapMap and dbSNP (Chinese population). Only SNPs, which may impact miRNA:LncRNA interaction according to the lncRNASNP database and limited the minimum allele frequency of SNPs in the Chinese population(CHB) > 5%, were selected. Finally, 16 SNPs were selected as candidate SNPs. GoldMag-Mini Whole Blood Genomic DNA Purification Kit (GoldMag Co. Ltd. Xi'an City, China) was used to extract DNA from the whole blood. DNA concentration was measured by NanoDrop 2000 (Gene Company Limited). Sequenom MassARRAY Assay Design 3.0 Software was used to design a Multiplexed SNP MassEXTEND assay [34]. Sequenom MassARRAY RS1000 was used for genotyping, and the data were managed using Sequenom Typer 4.0 Software [34, 35]. Primers used for this study are listed in Supplementary Table 1. Laboratory personnel were blinded to the genotyping results of all samples.

### Statistical analysis

Microsoft Excel and SPSS 21.0 statistical package (SPSS, Chicago, IL) were used to perform statistical analyses. All *p* values in this study were two-sided and differences were considered significant if *p*-value was less than 0.05. The genotypic distribution of each SNP in control subjects was tested for departure from Hardy–Weinberg Equilibrium (HWE) using an exact test. We compared the allele frequencies of cases and controls using the Pearson χ^2^ and Fisher exact tests. The association between genotypes and disease risks was evaluated by computing the odds ratio (OR) and their 95% confidence intervals (CI) using multiple logistic regression analysis. The most common genotype in the controls was used as the reference group. Associations between the selected SNPs and the risk of cervical cancer were assessed using genotypic model analysis (co-dominant, dominant, recessive, and log-additive) by SNP stats, a website software program available at http://bioinfo.iconcologia.net/snpstats/start.htm. We used the Haploview software package (version 4.2) and SHEsis software platform (http://analysis.bio-x.cn/myAnalysis.php) for analyses of linkage disequilibrium, haplotype construction, and genetic association at polymorphism loci. D′ value > 0.8 indicated that the related SNPs formed one block [36, 37]. In analysis of subtype and clinical stages, we examined associations separately for women with different squamous carcinomas, adenocarcinomas, and clinical stages I- II, III- IV, each compared to all controls.

## CONCLUSIONS

To the best of our knowledge, this is the first study to show miR-17-5p SNPs polymorphisms in cervical cancer. We demonstrate that four SNPs (rs217727, rs2862833, rs9931702, and rs9302648) of 16SNPs in the miR-17-5p are associated with cervical cancer risk. Large well-designed and population-based studies are warranted to confirm these findings.

## SUPPLEMENTARY MATERIALS TABLE


