# Supplementary material for: Association of the miR-17-5p variants with susceptibility to cervical cancer in a Chinese population
Source: Oncotarget. 2016 Sep 28;7(47):76647–55. doi: 10.18632/oncotarget.12299 (PMC5363537; doi:10.18632/oncotarget.12299)
Supplement: Supplementary file 1 [file oncotarget-07-76647-s001.pdf]

Association of the miR-17-5p variants with susceptibility to cervical cancer in a Chinese population

Supplementary Materials

Supplementary Table S1: Primers used for this study

SNP	1st-PCRP	2st-PCRP	UEP_SEQ
rs1048512	ACGTTGGATGTCCTTGCCTATATCCATTAG	ACGTTGGATGCAAGCCTTTAAAGAAGGAGC	gTTAAAGAAGGAGCTGTTCTGG
rs6659346	ACGTTGGATGTTAGCTGTTGTTGTTGGGGC	ACGTTGGATGTCTGGTAGGTTTTCCAGATA	ACAGGTTTGGAATCTTAATATC
rs11006369	ACGTTGGATGACGATCAGGAGTGTTTCAGC	ACGTTGGATGTGTTTTCGTTCTGCTGCAGG	gtGATGAAATCTGGATCCTTAATAAC
rs2862833	ACGTTGGATGTGGTAGGGGCTTGCTTTTTG	ACGTTGGATGTACCTTCTAAGGGATCCAAG	AGGGATCCAAGAAGCAT
rs3741216	ACGTTGGATGTCTCCACAACTCCAACCAGT	ACGTTGGATGAGAGATTCAAAGCCTCCACG	CCGTCCCTTCTGAATTT
rs217727	ACGTTGGATGACCGGCGACTCCATCTTCA	ACGTTGGATGTCAGCTCTGGGATGATGTGG	gGGCTGGTGGTCAACCGTCC
rs2839702	ACGTTGGATGCTGTTCCGATGGTGTCTTTG	ACGTTGGATGCACGTGTCGCTATCTCTAGG	GTGTCGCTATCTCTAGGTGAAGC
rs2067051	ACGTTGGATGCTCCTTGCTGCGCAATGTC	ACGTTGGATGGGAAGACAGGCAGTGCTCG	aAGTGCTCGGGGAGTTGCAGCAGGAC
rs2274062	ACGTTGGATGTCAAGGTACTGAGATGGGTG	ACGTTGGATGACACATTTAAGAGCCATTC	ccGAGCCATTCTTTTTACCACAT
rs9318375	ACGTTGGATGATTTCTGACCTCGTCAGTCC	ACGTTGGATGCTATCCTAATTCTTGTCCGC	TCTCCTGCTGATATGTTTGCC
rs2732044	ACGTTGGATGTTTCTCAGCTTCAGCACTAC	ACGTTGGATGCATATAGAACAGTACCCTCC	aACAACAACACAAACAAAATGA
rs1030389	ACGTTGGATGAATTTCATTCAGGCTTCCCG	ACGTTGGATGTTTTGCCGAAAGGCATCCTC	ATTCCCACACTGTAGCCAA
rs1045935	ACGTTGGATGGAGTACCAACTGTGTTCAAC	ACGTTGGATGGTGGTTTTATTGACCGTATC	TGGTTTTATTGACCGTATCATAACA
rs12902710	ACGTTGGATGAGTTTAGTGACGTGGAGCAG	ACGTTGGATGGTCTACAATTACCGTTACCG	CCGTTTTTAATAACCACTCAGG
rs9931702	ACGTTGGATGGTATGTAATGAGCAGTCTCC	ACGTTGGATGCTGACTCAGAAAGTAAGTGG	GCTAACTCATTGTTTCTTTTCTAAAAT
rs9302648	ACGTTGGATGACAGCACATTCTCTGGTGAC	ACGTTGGATGGCCAGCTGAAAAGAAGAGTC	ggAGAAGAGTCCTGACATG
